# Ion‐Induced Hydrophilic Switching Enables Nanostructure Morphology Control for Superior Nanoplasmonic Sensing

**DOI:** 10.1002/smll.202510984

**Published:** 2025-12-24

**Authors:** Chia‐Ming Yang, Chih‐Ching Ho, Aravind Satheesh, Chih‐Jen Yu, Nikhil Bhalla

**Affiliations:** ^1^ Institute of Electro‐Optical Engineering Chang Gung University Taoyuan City 33303 Taiwan; ^2^ Department of Electronic Engineering Chang Gung University Taoyuan City 33303 Taiwan; ^3^ Department of Materials Engineering Ming Chi University of Technology New Taipei City 243303 Taiwan; ^4^ Department of Neurosurgery Chang Gung Memorial Hospital at Linkou Taoyuan 33302 Taiwan; ^5^ Department of Electronics Engineering Ming‐Chi University of Technology New Taipei City 24301 Taiwan; ^6^ Nanotechnology and Integrated Bioengineering Centre (NIBEC) School of Engineering Ulster University 2‐24 York Street Belfast BT15 1AP UK

**Keywords:** Cahn–Hilliard, LSPR, nanostructures, sensors, substrate‐wettability

## Abstract

Controlling the morphology of dewetted ultrathin gold films is critical for achieving reproducible and high‐performance plasmonic sensors, yet scalable approaches remain limited. Localized surface plasmon resonance (LSPR) sensors rely on uniform metallic nanoislands whose morphology dictates optical sensitivity and signal reproducibility. Conventional solid‐state dewetting often produces non‐uniform nanostructures due to uncontrolled interfacial energy and adatom mobility, restricting wafer‐scale reproducibility. Here, a brief SF_6_ plasma pre‐treatment is introduced that induces ion‐mediated hydrophilic switching of glass surfaces, enhancing Au adatom mobility and promoting uniform nanoisland formation during thermal dewetting. The resulting structures exhibit reduced size dispersion and narrower interparticle gaps, yielding a 17.8% increase in refractive‐index sensitivity (from 80.79 ± 19.36 to 95.21 ± 6.56 nm RIU^−1^) with improved linearity and spectral reproducibility. Complementing these experiments, a modified Cahn–Hilliard phase‐field model embedding an explicit Au–substrate adhesion term (α) quantitatively reproduces the observed morphology and provides a predictive framework for tuning film evolution. This integrated experimental–theoretical‐simulation approach demonstrates that substrate‐wettability engineering via plasma activation offers a scalable, lithography‐free strategy for wafer‐level fabrication of uniform nanoplasmonic sensors, establishing a foundation for theory‐informed design of next‐generation plasmonic and photonic devices.

## Graphical abstract

SF_6_ treatment renders the glass substrate hydrophilic, enabling uniform nanoparticle distribution and resulting in superior LSPR sensing performance

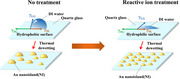

## 1 Introduction

Nanoplasmonic sensing platforms based on localized surface plasmon resonance (LSPR) have revolutionized label‐free optical detection by enabling rapid, ultrasensitive measurements of bio/chemical interactions.^[^ ^1^, ^2^, ^3^ ^]^ The sensing mechanism arises from the collective oscillation of conduction‐band electrons in metallic nanostructures,^[^ ^4^, ^5^ ^]^ such as gold,^[^ ^6^, ^7^ ^]^ or silver,^[^ ^8^, ^9^ ^]^ when illuminated at specific wavelengths. These oscillations generate intense local electromagnetic fields and give rise to sharp resonance peaks that shift in response to changes in the local refractive index.^[^ ^10^, ^11^ ^]^ The magnitude and sharpness of this shift is central to LSPR sensitivity which is intimately linked to the morphology, distribution, and uniformity of the underlying metallic nanostructures.^[^ ^12^, ^13^ ^]^

In recent years, localized surface plasmon resonance (LSPR) biosensing has emerged as one of the most powerful label‐free optical detection techniques for real‐time molecular interaction analysis. Its unique advantages, simple optical configuration,^[^ ^12^ ^]^ rapid response,^[^ ^14^ ^]^ and high sensitivity^[^ ^15^ ^]^ have led to diverse applications ranging from medical diagnostics and environmental monitoring to food safety.^[^ ^16^ ^]^ Ongoing research has focused on improving sensitivity,^[^ ^17^ ^]^ reproducibility, and integration with microfluidics^[^ ^7^ ^]^ to enable multiplexed and miniaturized sensing systems.

Despite extensive advances, one of the central challenges in the field remains achieving precise control over the morphology, spacing, and uniformity of metallic nanostructures across large areas. The optical performance of LSPR sensors is highly sensitive to nanoscale variations, yet reproducible fabrication at wafer scale is often hindered by stochastic film evolution and process variability. Therefore, developing scalable, lithography‐free methods capable of producing uniform and morphologically consistent nanostructures remains a key open problem in practical plasmonic sensor engineering.

Traditionally, achieving high‐performance plasmonic architectures has relied on top‐down fabrication techniques such as electron‐beam lithography^[^ ^18^, ^19^ ^]^ or nanoimprint lithography.^[^ ^20^, ^21^ ^]^ While these methods offer excellent spatial precision, they are inherently costly, time‐consuming, and limited in scalability. An alternative and increasingly attractive route involves solid‐state dewetting of ultrathin metal films, where a continuous film is thermally annealed to spontaneously reorganize into discrete nanoislands. This bottom‐up method enables rapid, large‐area fabrication of plasmonic nanostructures without the need for patterning, but suffers from one major drawback i.e. limited control over nanoisland size, spacing, and uniformity due to the complex interplay between film thickness, interfacial energy, and surface diffusion.

A key factor that governs the dewetting outcome is the wetting behavior of the metal film on the substrate.^[^ ^22^, ^23^ ^]^ The interfacial energy between the film and the substrate, often represented through a contact angle, determines the degree of film instability and the onset of mass redistribution. On pristine glass substrates, which exhibit moderate hydrophobicity,^[^ ^24^, ^25^ ^]^ ultrathin gold films tend to dewet unevenly, forming irregular, polydisperse structures with broad size distributions and inconsistent interparticle spacing. These morphological inconsistencies limit the reproducibility and sensitivity of LSPR‐based devices, especially when scaling up to wafer‐level processing.

It is also important to note that thermally dewetted Au nanoislands typically exhibit a polycrystalline nature, in contrast to the highly ordered or epitaxial nanoparticles obtained through wet‐chemical synthesis. While the latter can provide narrower plasmonic linewidths and lower damping due to enhanced crystalline order, such methods generally lack large‐scale spatial uniformity and integration compatibility. In contrast, our plasma‐assisted dewetting approach prioritizes wafer‐scale reproducibility, controllable interparticle spacing, and process scalability–key parameters for practical sensor fabrication. Moreover, the excellent optical performance achieved in this work, with an average refractive‐index sensitivity of 95.21 nm RIU^−1^ and low spectral variance, demonstrates that the resulting nanostructures possess sufficient optical quality for high‐performance LSPR sensing applications despite their polycrystalline nature.

In this work, we introduce an unexpectedly simple yet powerful approach to tune the dewetting process by modifying the substrate surface chemistry through SF_6_ plasma treatment. Sulfur hexafluoride, a commonly used fluorine‐rich gas in reactive ion etching, is known for its role in etching silicon‐based materials.^[^ ^26^, ^27^ ^]^ However, its influence on surface energy and wettability in the context of metal‐on‐glass dewetting remains poorly explored. Here, we show that brief exposure of glass substrates to SF_6_ plasma induces a hydrophilic switching effect, lowering the water contact angle from approximately 53° to 12°, and thereby dramatically decreasing increasing the solid–liquid interfacial surface energy. This ion‐induced modification is hypothesized to alter the density and composition of surface functional groups, possibly introducing polar moieties or removing organic contaminants that impede wettability.

The resulting increase in substrate hydrophilicity has a profound impact on the kinetics and morphology of dewetting. Gold atoms deposited on SF_6_‐treated surfaces exhibit higher mobility, reduced nucleation pinning, and more uniform mass redistribution during thermal annealing. Morphological analysis reveals that the mean nanoparticle diameter decreases, the aspect ratio improves, and edge‐to‐edge spacing narrows, all contributing to enhanced LSPR performance. Specifically, we observe a measurable increase in refractive index sensitivity and a reduction in spectral variance, making the resulting structures ideal candidates for biosensing applications that demand both sensitivity and reproducibility.

To complement our experimental observations and provide a theoretical underpinning, we develop a Cahn–Hilliard phase‐field model that simulates the dewetting dynamics as a function of surface mobility. The Cahn–Hilliard framework, widely used to study phase separation and interfacial phenomena,^[^ ^28^, ^29^, ^30^ ^]^ enables us to capture the temporal evolution of a conserved order parameter (gold concentration) under the influence of thermodynamic and kinetic forces. In our implementation, the substrate surface energy enters implicitly through the mobility parameter, which we tune to reflect the enhanced diffusion observed on SF_6_‐treated glass. The model successfully reproduces key features of the experimental morphology, including reduced island size, increased island density, and tighter spacing distributions.

Importantly, this experiment–model synergy allows us to extract a quantitative estimate of the relative enhancement in surface mobility induced by plasma treatment–information that would be difficult to obtain through experimental methods alone. Furthermore, the phase‐field approach provides predictive capability, allowing us to explore parameter spaces such as annealing time, film thickness, and temperature to optimize nanostructure formation in future studies.

Our results demonstrate that ion‐induced surface energy modulation offers a powerful, scalable, and lithography‐free strategy to direct metal film dewetting toward desirable morphological outcomes. Beyond the fundamental interest in surface wetting and thin film dynamics, this approach has immediate implications for the rational design of nanoplasmonic sensors, where uniformity, sensitivity, and scalability are of paramount importance. The use of SF_6_ plasma is particularly attractive because it is compatible with standard microfabrication workflows^[^ ^1^ ^]^ and can be easily integrated into existing process lines. These findings open new avenues for the scalable fabrication of next‐generation LSPR‐based sensors and nanophotonic devices.

## 2 Results and Discussion

**Figure 1:**
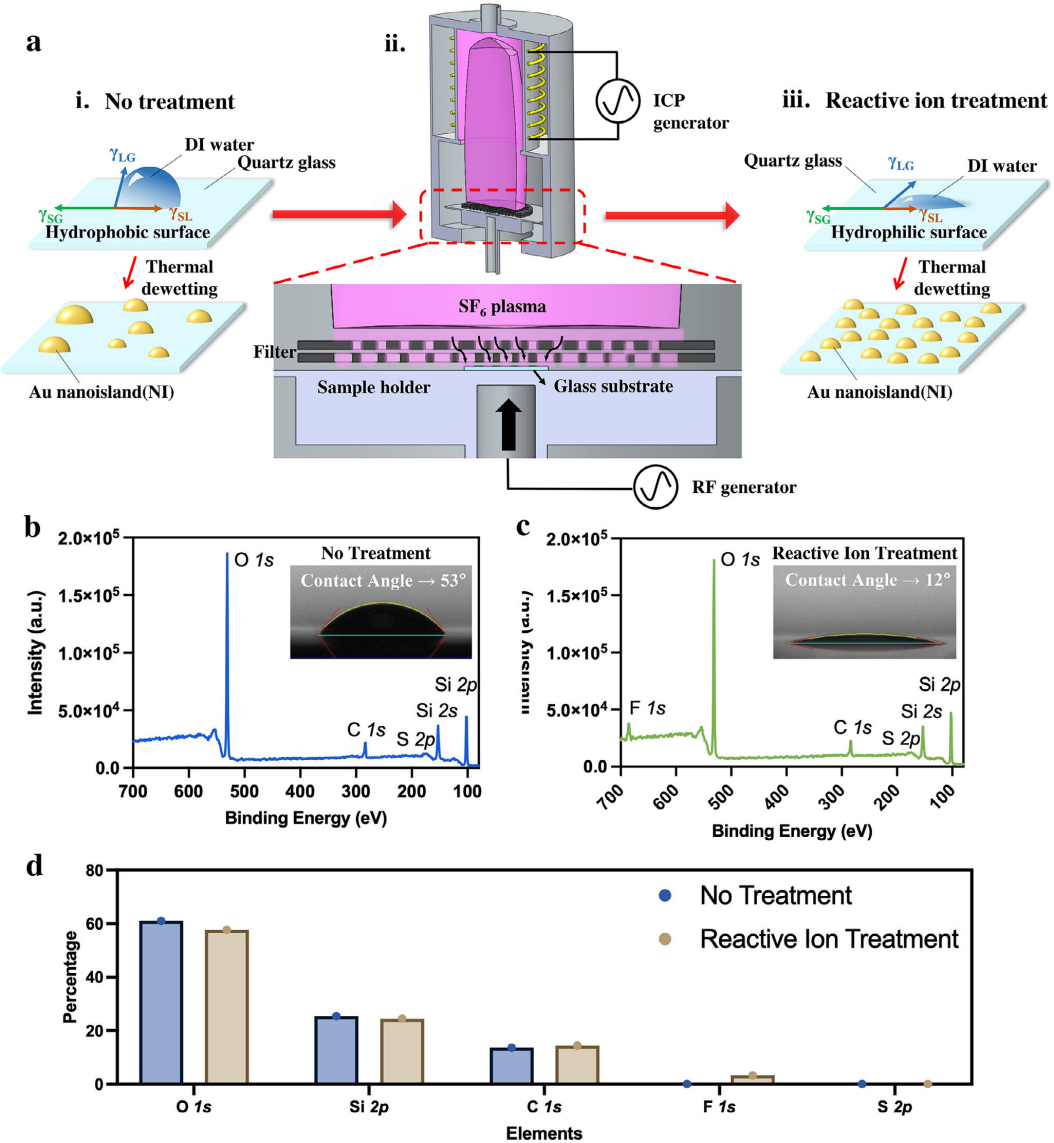
Schematic and characterization: a) schematic showing experimental setup of our study and its outcome. b) Untreated quartz glass XPS and contact angle (≈53°) results, c) reactive ion treated glass XPS and contact angle (≈12°) results that promotes uniform dewetting and d) elemental ratio analysis.

**Figure** **1**a shows the schematic of our experiments and its outcome. Essentially, the untreated glass substrates remain moderately hydrophobic. Following SF_6_ plasma filterd reactive ion treatment, hydrophilic functional groups are introduced onto the glass. The initial influence of SF_6_ plasma treatment on substrate wettability was assessed using contact angle measurements and X‐ray Photoelectron Spectroscopy (XPS). Untreated quartz glass exhibited a contact angle of approximately 53° (Figure 1b) and no Fluorine (F) element was found in the XPS results, whereas plasma‐treated glass showed a markedly reduced contact angle of 12° (Figure 1c) and a obvious peak for F*1s*. The F*1s* peak centered at 686.5 eV indicates the formation of inorganic Si–F/Si–O–F oxyfluoride bonds rather than hydrophobic CF_x_ species, as no C*1s* components corresponding to CF, CF_2_, or CF_3_ were detected. (Detailed XPS analysis and discussion are provided in the Supporting Information Figure S1.) AFM analysis (Supporting Information Figure S2) revealed no detectable change in surface roughness before and after the filtered SF_6_ plasma treatment, confirming negligible ion bombardment or etching effects. Therefore, the observed enhancement is attributed to fluorine‐induced surface chemistry rather than roughness modification, with detailed AFM results provided in the Supporting Information. This drastic reduction in θ reflects a significant increase in hydrophilicity and, consequently, a decrease in surface energy between liquid and the glass substrate. The thermodynamic basis for this effect can be described by Young's equation:^[^ ^22^, ^23^ ^]^

(1) $$\gamma_{SG}=\gamma_{SL}+\gamma_{LG}\cos\theta$$

which may be rearranged as:

(2) $$\gamma_{SL}=\gamma_{SG}-\gamma_{LG}\cos\theta$$

where, γ_*SG*_ is the solid–gas surface energy, γ_*SL*_ is the solid–liquid interfacial energy, γ_*LG*_ is the liquid–gas surface tension and θ is the contact angle.

According to Young's equation, a decrease in the water contact angle corresponds to a reduction in the apparent solid–liquid interfacial energy (γ_*SL*_) for the water–glass system. While this relation provides a convenient descriptor of wettability, it does not directly quantify the adhesion energy between gold and glass, since metal–substrate interactions depend on additional factors such as interfacial bonding, electronic density overlap, and chemical termination. Therefore, the correlation between hydrophilicity and Au–glass adhesion in this work should be viewed as qualitative. The SF_6_ plasma treatment likely alters the surface composition and polar functionality of glass, which modifies both the surface energy landscape and the adatom diffusion kinetics. Within this context, a reduced effective adhesion parameter (α) in our phase‐field model represents the experimentally observed enhancement of Au adatom mobility on treated substrates, rather than a literal decrease in thermodynamic adhesion energy.

Consequently, Au adatoms experience a smoother energy landscape and can diffuse more efficiently during thermal annealing as also dipicted in Figure 1a(iii), forming smaller and denser Au NIs. Figure 1d summarizes the elements identified on the glass substrates with and without SF_6_ plasma treatment in Figure 1b,c essentially confirming that the fluorine element on the surface is responsible for the observed hydrophilicity.

**Figure 2:**
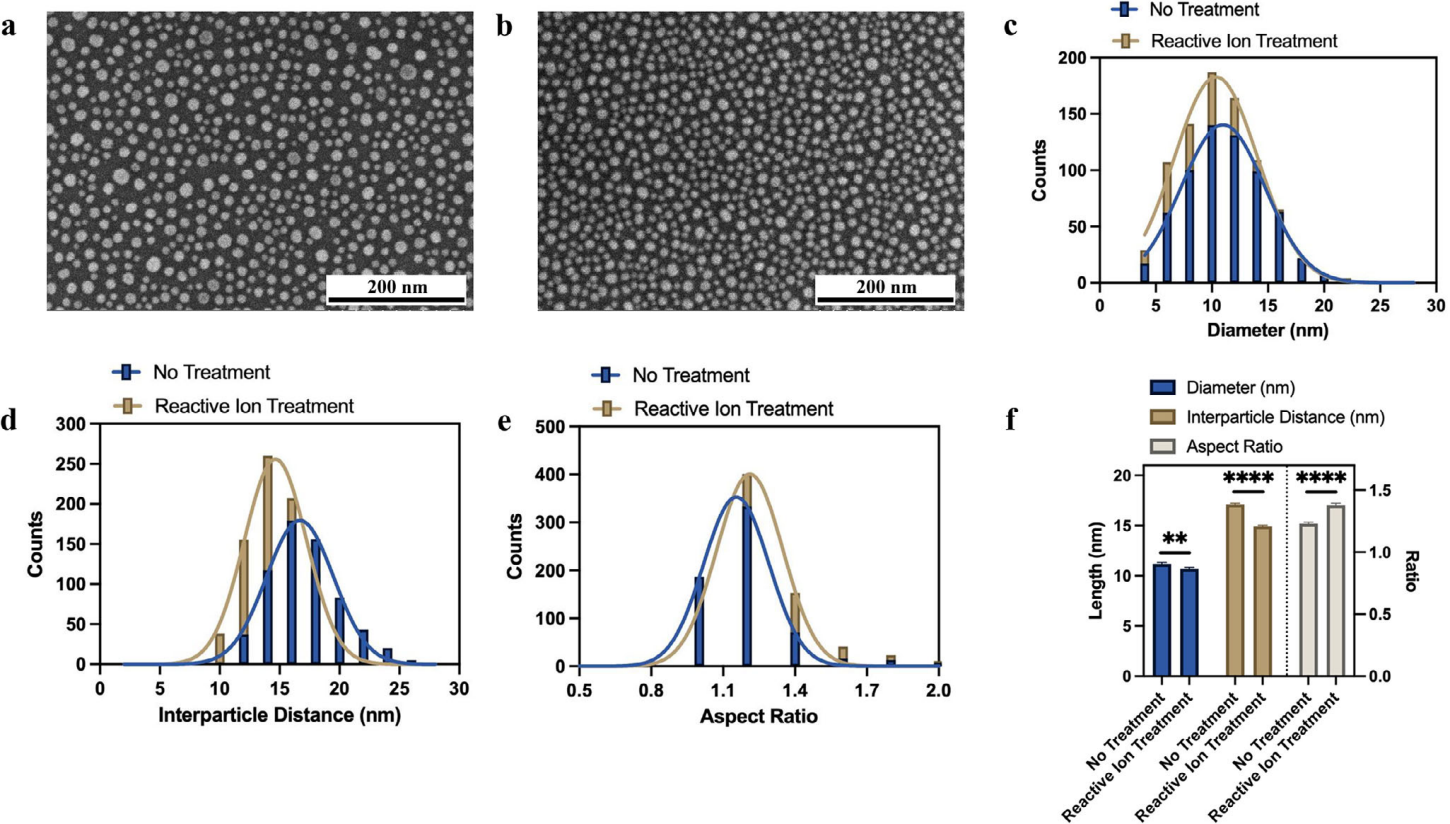
Experiment: SEM image of a)untreated and b) treated Au nanoislands, and there, c) size distribution, d) interparticle distance, e) aspect ratio, and f) statistical comparison, showing improved uniformity and reduced variation after treatment.

The impact of plasma treatment on dewetting morphology of Au NIs is clearly seen in high‐resolution SEM images (**Figure** **2**). On untreated glass (Figure 2a), NIs form with larger size and interparticle distance. The limited diffusion length of Au adatoms leads to clustering, void formation, and poor uniformity. These features are symptomatic of a system with high γ_*SL*_, where strong interfacial pinning suppresses mass redistribution. By contrast, plasma‐treated substrates (Figure 2b) show an improvement in NIs morphology. The NIs are smaller (Figure 2c), less spaced (Figure 2d), and less rounded in shape (Figure 2e). Figure 1f summarizes these comparisons. The Au NI size decrease from 11.23 to 10.73 nm, interparticle distance reduce from 17.11 nm to 14.96 nm and aspect ratio increase from 1.23 to 1.38. In Figure 2f, the results are also statistically compared with Welch's *t* test with P value < 0.0001 (number of * indicate the level of significance difference). This regularity in the reactive ion treated surfaces arises because the enhanced hydrophilicity lowers γ_*SL*_, which facilitates efficient adatom migration and nucleation. The resulting morphology suppresses coarsening instabilities and produces nanoscale features that are far more reproducible across the substrate from batch to batch fabrication (discussed later). Such uniformity is crucial for plasmonic sensing, where consistent hot‐spot localization depends sensitively on island density and spacing,^[^ ^31^, ^32^ ^]^ also higher aspect ratio will benefit the sensitivity.^[^ ^33^, ^34^ ^]^

To establish a mechanistic framework for these observations, we develop our own numerical model by using a Cahn–Hilliard (CH) phase‐field model. This approach describes the temporal evolution of a conserved order parameter *c*(**r**, *t*), representing the local Au concentration on the substrate. The model is based on the minimization of a free energy functional. In order to discuss the interaction between the glass substrates and Au atoms, we define the free energy functional as

(3) $$F\left[c\right]=\int_{\Omega }\left(f\left(c\right)+\frac{\varepsilon^{2}}{2}{\left|\nabla c\right|}^{2}+f_{sub}\left(c\right)\right)\;dr$$

where, $f\left(c\right)=\frac{1}{4}{\left(c^{2}-1\right)}^{2}$ is the bulk free energy (double‐well potential), ϵ controls the interfacial width and line tension, $f_{sub}\left(c\right)=\frac{1}{2}\alpha c^{2}$ is the quadratic substrate interaction term, This form is consistent with previous phase‐field treatments, where the wall free energy is often expanded as a polynomial up to the second order with respect to the order parameter at the substrate,^[^ ^35^ ^]^ α parameterizes the strength of Au–substrate adhesion.

The corresponding chemical potential is derived as the variational derivative of *F*:

(4) $$\mu =\frac{\delta F}{\delta c}=c^{3}-c-\varepsilon^{2}\nabla^{2}c+\alpha c.$$

The governing CH equation for the temporal evolution of *c* is then:

(5) $$\frac{\partial c}{\partial t}=\nabla \cdot \left(M\nabla \mu \right)$$

which expands to:

(6) $$\frac{\partial c}{\partial t}=\nabla \cdot \left[M\nabla \left(c^{3}-c-\varepsilon^{2}\nabla^{2}c+\alpha c\right)\right].$$

Here, plasma treatment decreased α for weaker Au–substrate interaction and enhanced surface mobility of adatoms (enhanced diffusivity).

For computational efficiency, the Laplacian operator was treated in Fourier space. Letting $\hat{c}=F\left[c\right]$, $\hat{\mu }=F\left[\mu \right]$ and defining $k^{2}=k_{x}^{2}+k_{y}^{2}$, the Laplacian in Fourier space becomes:

(7) $$F\left[\nabla^{2}c\right]=-k^{2}\hat{c}$$

Thus, the time‐stepping update for $\hat{c}$ in Fourier space is:

(8) $$\hat{c}\left(t+\Delta t\right)=\hat{c}\left(t\right)-\Delta t\cdot M\cdot k^{2}\cdot \hat{\mu }$$

Finally, the inverse Fourier transform is used to retrieve the updated concentration field in real space:

(9) $$c\left(t+\Delta t\right)=F^{-1}\left[\hat{c}\left(t+\Delta t\right)\right]$$

**Figure 3:**
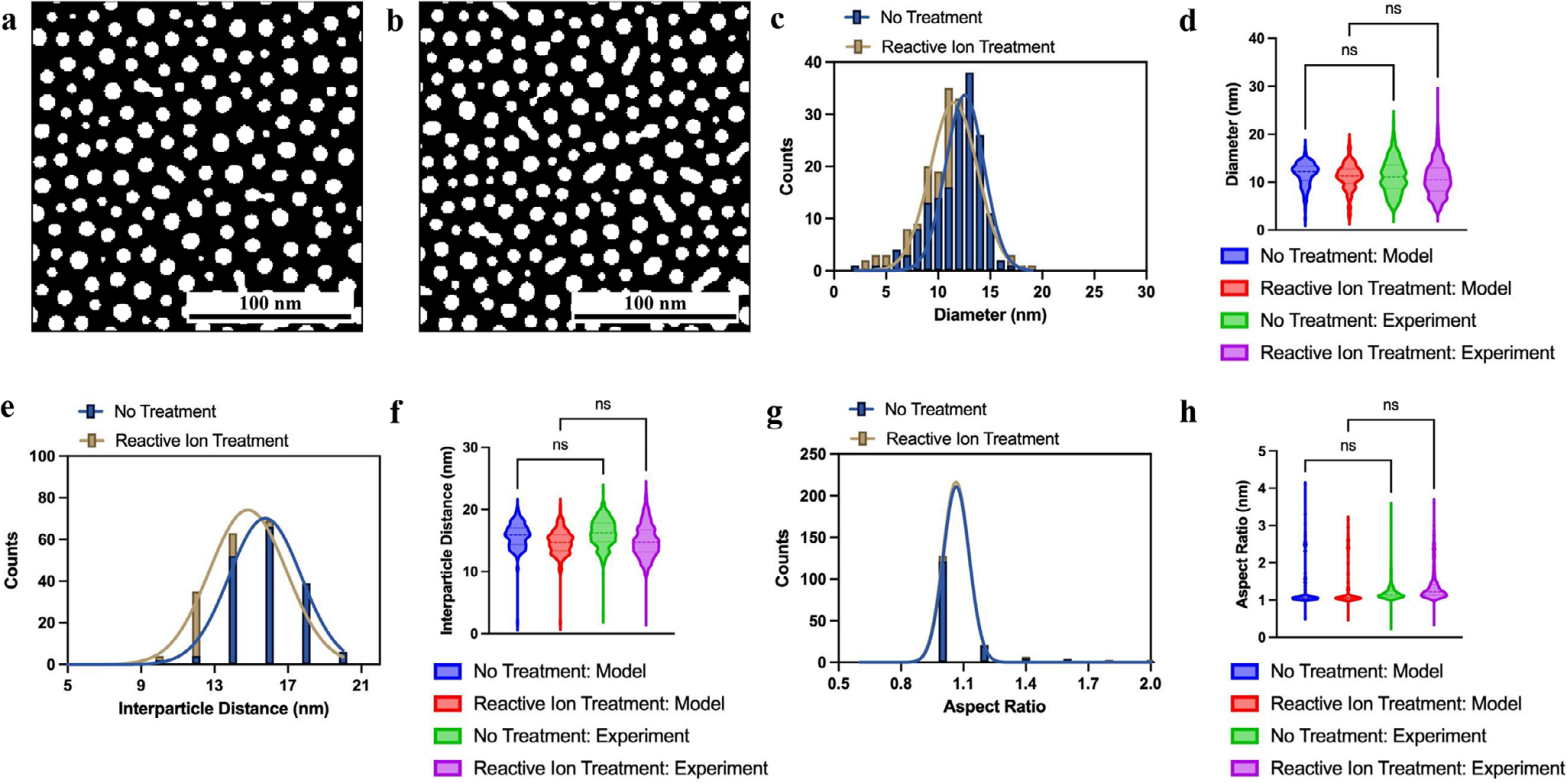
Modeling: a) Simulated Au nanoislands without treatment and b) after reactive ion treatment, c) NI size distribution, e) interparticle distance distribution, g) aspect ratio distribution, and d,f,h) corresponding violin plots, showing good agreement between simulation and experimental data.

The simulated morphologies in **Figure** **3** validate this framework. Figure 3a (untreated case, higher α) yield less NIs with larger size, closely resembling the experimental SEMs of untreated glass (Figure 2a). Figure 3b (treated case, reduced α) evolve into smaller and denser NIs, reproducing the uniform morphologies of ion reactived‐treated substrates (Figure 2b). This close agreement between simulation and experiment confirms that weakened interfacial adhesion is the key driver of uniform dewetting.

The statistical comparisons in Figure 3c–h further support this conclusion. The simulated NIs size distributions (Figure 3c) exhibit strong consistency with experimental measurements, both showing only a slight reduction in average size from 11.77 nm (untreated) to 11.21 nm (treated). Violin plots of (Figure 3d) reveal same trend between model and experiment, highlighting the predictive reliability of the simulation. Similarly, the interparticle distance distributions (Figure 3e) demonstrate closer spacing for the treated case, with the average distance decreasing from 15.81 nm (untreated) to 14.78 nm (treated), in agreement with experimental data. The corresponding violin plots (Figure 3f) confirm that both model and experiment converge on the same interparticle distance values without statistically significant differences. Aspect ratio distributions (Figure 3g) remain narrowly centered around unity in both treated and untreated cases, with simulated averages of 1.26 (untreated) and 1.33 (treated), consistent with nearly isotropic island growth. This is further substantiated by violin plots in Figure 3h, where both model and experiment show similar distributions and no significant differences. Together, these results establish that the model not only reproduces overall morphological trends but also quantitatively captures the statistical descriptors of NI ensembles under different interfacial conditions.

**Figure 4:**
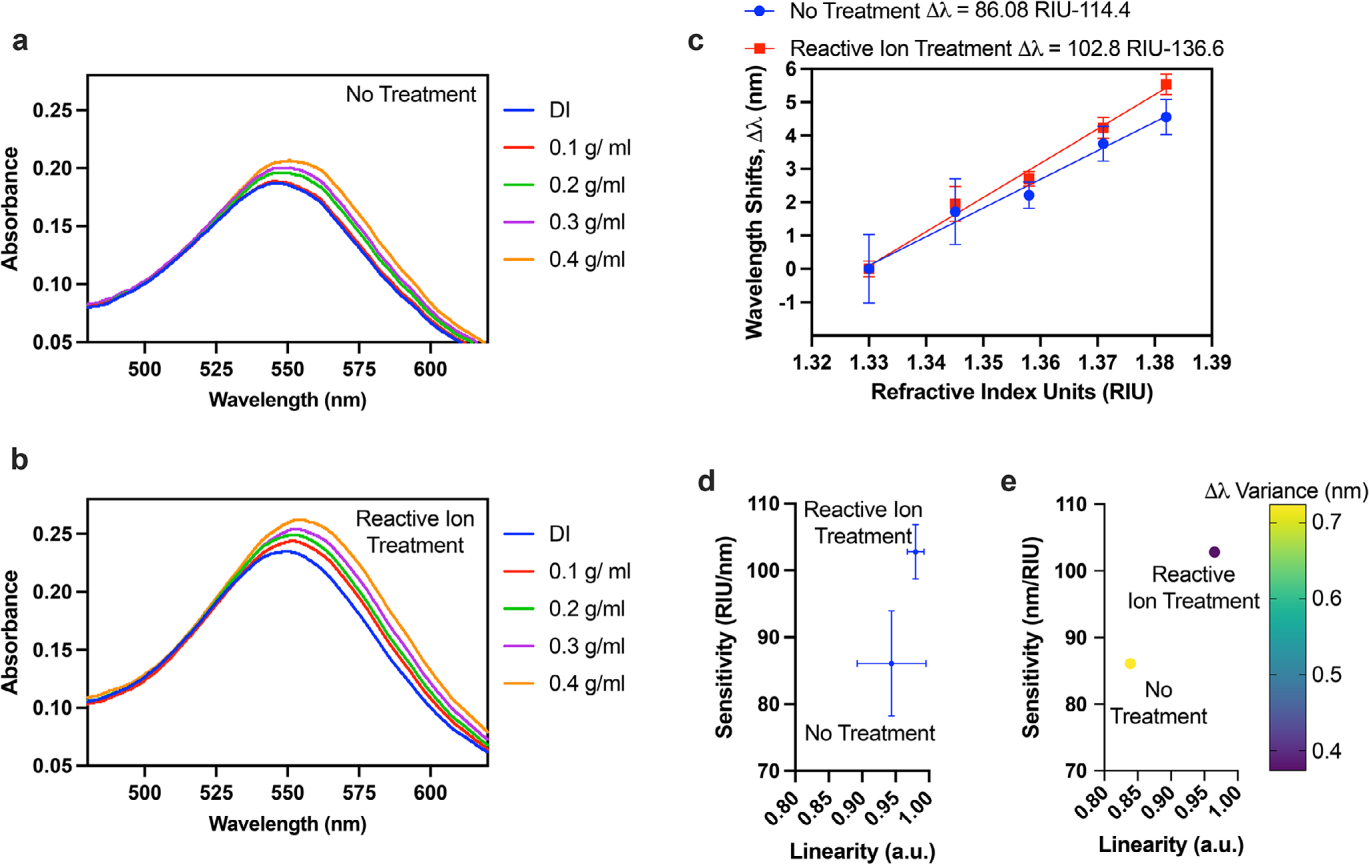
Optical and sensing characteristics: a) Refractive index response of untreated Au nanoislands and b) reactive ion treated samples, c) Refractive index sensitivity of untreated and treated sample, d) sensitivity and linearity comparison, and e) variance analysis, showing improved sensitivity, linearity, and reproducibility after reactive ion treatment.

The structural improvements resulting from plasma treatment have direct implications for plasmonic sensing. LSPR measurements were performed using glucose solutions of varying refractive indices. The absorbance spectra for untreated NIs (**Figure** **4**a) show modest intensity (∼0.20) with broad features, consistent with fewer resonant particles and irregular morphology. In contrast, ion reactive‐treated NIs (Figure 4b) exhibit stronger absorbance (∼0.25), due to the denser packing and smaller interparticle spacing that enable more efficient coupling of incident light to localized plasmon modes.

Figure 4c quantifies this improvement in terms of refractive index sensitivity. The untreated NIs exhibit a sensitivity of 86 nm RIU^−1^, while plasma‐treated nanoislands achieve 102 nm RIU^−1^. This ∼19% enhancement is accompanied by smaller error bars for the treated case, reflecting superior reproducibility, and the obtained sensitivity is comparable to, or even exceeds, values reported in previous LSPR biosensing studies using Au nanoislands with similar film thickness.^[^ ^12^, ^13^, ^36^, ^37^ ^]^ In addition to sensitivity, a detailed comparison of interparticle spacing, and uniformity with previously reported dewetting‐based LSPR sensors is provided in the Supporting Information (Table S1). Overall, our SF_6_ plasma–treated substrates exhibit smaller feature sizes, narrower spacing, and improved uniformity while maintaining high sensitivity.

Statistical evaluation across substrate from same batch (Figure 4d) further highlights the improved sensitivity and linearity after the reaction ion treatment. Additionally, the reduced standard deviation demonstrates that reactive ion treatment not only improves sensitivity but also ensures consistency various locations on the substrate, an essential requirement for developing homogeneous LSPR substrates for biosensing applications. Figure 4e further substantiates these findings by the variance in resonance peak shifts (Δλ variance), which was calculated from the standard deviation of the residuals. Reactive ion–treated substrates show higher sensitivity and superior linearity, accompanied by lower Δλ variance, confirming that this treatment improves both performance and reproducibility of the LSPR response.

LSPR sensitivity and figure‐of‐merit depend on a co‐optimization of particle size, shape,^[^ ^38^ ^]^ interparticle gap,^[^ ^32^ ^]^ aspect ratio,^[^ ^33^, ^34^ ^]^ and damping effects,^[^ ^39^ ^]^ rather than size alone.^[^ ^40^ ^]^ Our SF_6_‐assisted dewetting approach enables this tunability through controlled process parameters, as supported by prior studies demonstrating gap‐dominated sensitivity and non‐monotonic size dependence in LSPR sensing.

**Figure 5:**
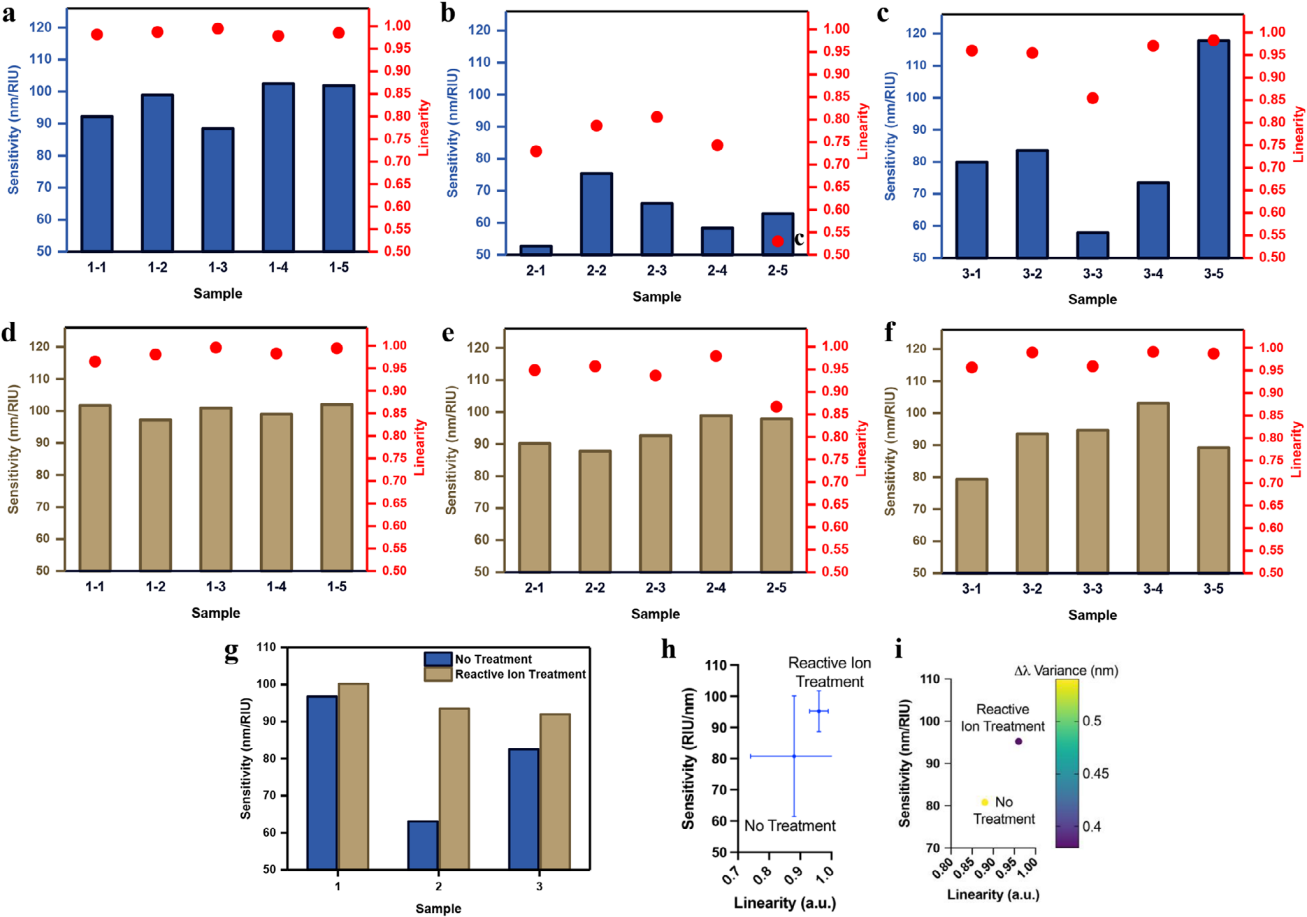
Reproducibility and Stability: a–c) Sensitivity and linearity of untreated glass across three samples, d–f) corresponding results after reactive ion treatment, g) averaged sensitivity comparison, h) sensitivity versus linearity, and i) variance analysis, demonstrating improved reproducibility and stability after treatment.

To further evaluate reproducibility and temporal stability under large‐scale fabrication, refractive index measurements were performed at five locations on each of three chips (15 in total). Untreated samples showed large fluctuations in sensitivity (96.77, 63.08, and 82.53 nm RIU^−1^) and unstable linearity (**Figure** **5**a–c), whereas treated chips exhibited more consistent responses (100.19, 93.48, and 95.21 nm RIU^−1^) with improved stability (Figure 5d–f). Averaging all measurements (Figure 5g,h) revealed a sensitivity increase from 80.79 ± 19.36 to 95.21 ± 6.56 nm RIU^−1^ and a linearity improvement from 0.88 ± 0.14 to 0.97 ± 0.03 after treatment, with smaller error bars further confirming enhanced uniformity. The reproducibility is reinforced in Figure 5i, where the Δλ variance—calculated from the standard deviation of the residuals—remains lower for treated samples. Collectively, these results demonstrate that reactive ion treatment yields more uniform Au NIs, enhancing sensitivity while ensuring reproducibility, an essential requirement for scalable LSPR biosensing substrates.

Furthermore, we emphasise that the observed enhancement in sensitivity and uniformity was achieved using a single, rapid SF_6_ plasma treatment fully compatible with standard microfabrication workflows. This highlights the scalability and practicality of the approach for wafer‐level integration, beyond isolated nanoscale demonstrations. In addition, our work introduces a substrate‐coupled theoretical framework based on a modified Cahn–Hilliard model that explicitly accounts for the Au–substrate adhesion term (α). The excellent agreement between simulation and experiment underscores the predictive capability of this model, enabling theory‐informed process optimization and reciprocal validation of future experiments. Together, these aspects establish a powerful experimental–theoretical synergy that benchmarks our results against the current state of the art and provides a pathway toward rational, scalable nanoplasmonic device design.

## 3 Conclusion

Our findings open a promising pathway toward reproducible, high‐performance plasmonic biosensing by exploiting ion‐induced hydrophilic switching of quartz substrates. SF_6_ plasma pretreatment transforms the surface from hydrophobic to hydrophilic, enhancing Au adatom mobility and reducing interfacial adhesion during dewetting. Consequently, Au nanoislands with smaller diameters, narrower spacing, and improved uniformity are obtained, directly benefiting localized surface plasmon resonance (LSPR) sensing.

Experimentally, plasma‐treated nanoislands achieved a 17.8% increase in refractive index sensitivity (from 80.79 ± 19.36 to 95.21 ± 6.56 nm RIU^−1^), accompanied by reduced spectral variance and enhanced linearity, underscoring superior reproducibility across wafer‐scale fabrication. Complementary Cahn–Hilliard simulations further validated that increased adatom diffusion and weakened Au–substrate interaction drive these uniform morphologies, providing predictive insight into nanostructure evolution.

In summary, substrate wettability engineering represents a lithography‐free, scalable strategy for fabricating stable and sensitive plasmonic substrates. This integrated experimental–theoretical framework not only advances the reliability of LSPR sensors but also establishes a foundation for designing next‐generation nanoplasmonic devices where sensitivity, reproducibility, and scalability are essential.

## 4 Experimental Section

### 4.1 Au Nanoisland Fabrication

Quartz glass substrates (Ruilong, Taiwan) were first cleaned sequentially with acetone, methanol, and deionized (DI) water, followed by drying at 120 °C for 5 min to remove residual moisture. Subsequently, reactive ion treatment was performed using an inductively coupled plasma reactive ion etching (ICP‐RIE) system with SF_6_ plasma. During the treatment, a filter was placed between the plasma and the substrate to suppress ion bombardment, allowing predominantly radicals to reach the surface and react chemically. The process conditions were maintained at a chamber pressure of 100 mTorr, SF_6_ flow rate of 20 sccm, ICP power of 150 W, RF bias power of 10 W, and a treatment duration of 5 min. Afterwards, a 4 nm gold film was thermally evaporated onto the substrates using a thermal evaporation system. Finally, the samples were subjected to rapid thermal annealing (RTA) at 650 °C for 1 min to induce the formation of gold nanoislands.

### 4.2 Contact Angle Measurement

Contact angle measurements between the quartz glass substrate and deionized (DI) water were performed using an FTA 22AUC03 system (The Imaging Source, Germany). A 1 μL droplet of DI water was dispensed onto the substrate surface with a pipette, and images were captured and determined using FTA 32 software.

### 4.3 X‐Ray Photoelectron Spectroscopy (XPS)

Analysis was conducted on a PHI 5000 VersaProbe III system (ULVAC‐PHI, Japan) equipped with a vacuum system (⩽ 6.7 × 10^−8^ Pa). The analysis area was tunable from ⩽ 10 μm to 1400 μm. Measurements were carried out within a few hours after plasma treatment to minimize signal decay over time.

### 4.4 Scanning Electron Microscopy (SEM)

Images were obtained using a HITACHI SU‐8220 (Hitachi, Japan) field‐emission SEM at an accelerating voltage of 10.0 kV, and a magnification of 200 000×. The system supports accelerating voltages up to 30 kV with a magnification range of 30× to 500 000×.

### 4.5 Optical Measurement

Absorbance spectra were measured using a halogen light source (HL‐2000, Ocean Optics, USA) vertically incident from the bottom side. The source provided a wavelength range of 360–2400 nm. The transmitted light signal was collected from the top side and analyzed by a spectrometer (USB‐4000, Ocean Optics, USA). The spectrometer covered a wavelength range of 200–1100 nm with a minimum spectral resolution of 0.21 nm.

### 4.6 Stastistics Analysis

The refractive index (RI) sensitivity was determined by performing Gaussian fitting of the absorbance spectra using Origin software (OriginLab, USA). The peak positions were extracted by fitting the spectral region near the resonance, and the spectral shifts under different refractive indices were plotted to obtain the sensitivity curve. For the analysis of Au nanoislands (NIs), scanning electron microscopy (SEM) images were first processed using ImageJ software (NIH, USA) to apply thresholding. The processed images were subsequently analyzed with a custom MATLAB script (MathWorks, USA) to quantify the average diameter, aspect ratio (AR), and interparticle distance distribution of the Au NIs.

### 4.7 Cahn–Hillard(CH) Phase Field Simulation

Old Thermal dewetting was simulated using a 2D Cahn–Hilliard phase field model in MATLAB. The governing free energy functional contained bulk double‐well, gradient, and substrate interaction terms, and was solved by a Fourier spectral method with periodic boundary conditions to mimic an extended thin film. Key simulation parameters included adhesion term α, the mobility M, time step dt, interface width ϵ, and initial concentration c. NI morphology was quantified through binary segmentation and connected component analysis to extract size, aspect ratio and interparticle distance.

### 4.8 Determination of Model Parameters

The adhesion term $\frac{1}{2}\alpha c^{2}$ in the free‐energy functional was formulated following Wang and Nestler's work,^[^ ^35^ ^]^ where the wall free energy is expressed as

(10) $$r\left(\phi_{s}\right)=\omega_{0}+\omega_{1}\phi_{s}+\frac{1}{2}\omega_{2}\phi_{s}^{2}.$$

Here, ω_2_ defines the curvature of the wall energy and thereby characterizes the interfacial adhesion strength between the film and the substrate. In our simplified model, the parameter α directly corresponds to this curvature term and retains the physically relevant contribution to the chemical‐potential gradient driving the Cahn–Hilliard dynamics. The constant (ω_0_) and linear (ω_1_) components were neglected because they only shift or tilt the energy landscape without influencing the evolution.

In practice, α was treated as an effective fitting parameter to match the experimentally observed nanoisland size and spacing for untreated and plasma‐treated samples. Lower values of α produced smaller, denser nanoislands, consistent with enhanced adatom mobility on SF_6_‐treated substrates. The mobility *M* was held constant across all simulations, corresponding to the fixed annealing conditions (temperature and duration) used experimentally. Therefore, morphology variations are attributed primarily to changes in the adhesion parameter α, while *M* represents the experimental thermal diffusion environment.

## Conflict of Interest

The authors declare no conflict of interest.

## Supporting information



Supporting Information

## Data Availability

The data that support the findings of this study are available from the corresponding author upon reasonable request.

## References

[1] J. Waitkus , Y. Chang , L. Liu , S. V. Puttaswamy , T. Chung , A. M. M. Vargas , S. J. Dollery , M. R. O'Connell , H. Cai , G. J. Tobin , et al., Adv. Mater. Interfaces 2023, 10, 2201261. 37091050 10.1002/admi.202201261 PMC10121183

[2] P. Pereira‐Silva , D. I. Meira , A. Costa‐Barbosa , D. Costa , M. S. Rodrigues , J. Borges , A. V. Machado , A. Cavaleiro , P. Sampaio , F. Vaz , Nanomaterials 2022, 12, 1526. 35564234 10.3390/nano12091526 PMC9102245

[3] S. Kastner , A.‐K. Dietel , F. Seier , S. Ghosh , D. Weiß , O. Makarewicz , A. Csáki , W. Fritzsche , Small 2023, 19, 2207953. 10.1002/smll.202207953 37093195

[4] R. L. Gieseking , Mater. Horiz. 2022, 9, 25. 34608479 10.1039/d1mh01163d

[5] W. Ou , B. Zhou , J. Shen , C. Zhao , Y. Y. Li , J. Lu , Iscience 2021, 24, 2. 10.1016/j.isci.2020.101982 PMC7820137 33521596

[6] T. Lamtha , U. Waiwijit , K. Duangkanya , T. Lertvanithphol , R. Amarit , K. Tantiwanichapan , A. Sathukarn , C. Chananonnawathorn , W. Hincheeranan , K. Dhanasiwawong , K. Choowongkomon , T. Sritrakul , O. Boodde , M. Sukmak , M. Horprathum , Sens. Actuators, A: Physical 2025, 382, 116165.

[7] J.‐S. Chen , P.‐F. Chen , H. T.‐H. Lin , N.‐T. Huang , Analyst 2020, 145, 7654. 32966364 10.1039/d0an01201g

[8] P. P. A. Suthanthiraraj , A. K. Sen , Biosens. Bioelectron. 2019, 132, 38. 30851494 10.1016/j.bios.2019.02.036

[9] Y. Yang , J. Murray , J. Haverstick , R. A. Tripp , Y. Zhao , Sens. Actuators, B 2022, 359, 131604. 10.1016/j.snb.2022.131604 PMC8857771 35221531

[10] J. W. Ha , Appl. Spectrosc. Rev. 2023, 58, 346.

[11] M. Li , S. K. Cushing , N. Wu , Analyst 2015, 140, 386. 25365823 10.1039/c4an01079e PMC4274271

[12] H. T.‐H. Lin , C.‐K. Yang , C.‐C. Lin , A. M.‐H. Wu , L. A. Wang , N.‐T. Huang , Nanomaterials 2017, 7, 100. 28468325 10.3390/nano7050100 PMC5449981

[13] N. Bhalla , A. Jain , Y. Lee , A. Q. Shen , D. Lee , Nanomaterials 2019, 9, 1530. 31717894 10.3390/nano9111530 PMC6915419

[14] S. S. Aćimović , H. Šípová , G. Emilsson , A. B. Dahlin , T. J. Antosiewicz , M. Käll , Light: Sci. Appl. 2017, 6, e17042. 30167285 10.1038/lsa.2017.42 PMC6062313

[15] S. Unser , I. Bruzas , J. He , L. Sagle , Sensors 2015, 15, 15684. 26147727 10.3390/s150715684 PMC4541850

[16] M. E. Hamza , M. A. Othman , M. A. Swillam , Biology 2022, 11, 621.35625349

[17] T. Xu , Z. Geng , Biosens. Bioelectron. 2021, 174, 112850. 33309521 10.1016/j.bios.2020.112850

[18] M. Hentschel , J. Karst , H. Giessen , Adv. Opt. Mater. 2020, 8, 2000879.

[19] H. Ahmed , M. Wu , M. Stepanova , J. Vac. Sci. Technol., B 2023, 41, 2.

[20] V. Gupta , S. Sarkar , O. Aftenieva , T. Tsuda , L. Kumar , D. Schletz , J. Schultz , A. Kiriy , A. Fery , N. Vogel , et al., Adv. Funct. Mater. 2021, 31, 2105054.

[21] M. F. S. Shahidan , J. Song , T. D. James , A. Roberts , Nanoscale Adv. 2020, 2, 2177. 36132510 10.1039/d0na00038h PMC9416936

[22] C. V. Thompson , Annu. Rev. Mater. Res. 2012, 42, 399.

[23] P. Potejanasak , S. Duangchan , Crystals 2020, 10, 533.

[24] J. Y. Kim , N. B. Brauer , V. Fakhfouri , D. L. Boiko , E. Charbon , G. Grutzner , J. Brugger , Opt. Mater. Express 2011, 1, 259.

[25] J. Xu , C. Wang , T. Wang , Y. Wang , Q. Kang , Y. Liu , Y. Tian , RSC Adv. 2018, 8, 11528. 35542819 10.1039/c7ra13095c PMC9079126

[26] P. Arora , T. Nguyen , A. Chawla , S.‐K. Nam , V. M. Donnelly , J. Vac. Sci. Technol., A 2019, 37, 6.

[27] S. Cheon , D. S. Jeong , J.‐K. Park , W. M. Kim , T. S. Lee , H. Lee , I. Kim , J. Phys. D: Appl. Phys. 2018, 51, 125102.

[28] Y. Feng , Y. Feng , G. Iyer , J.‐L. Thiffeault , J. Nonlinear Sci. 2020, 30, 2821.

[29] J. Kim , S. Lee , Y. Choi , S.‐M. Lee , D. Jeong , Math. Probl. Eng. 2016, 2016, 9532608.

[30] J. Shin , Y. Choi , J. Kim , Math. Probl. Eng. 2019, 2019, 1710270.

[31] S. Jia , A. Ma , H. Dong , S. Xia , Sensors 2022, 22, 9075. 36501777 10.3390/s22239075 PMC9739458

[32] A. Bonyár , Biosensors 2021, 11, 527.34940284

[33] C. Du , W. Yang , S. Peng , D. Shi , Phys. Chem. Chem. Phys. 2019, 21, 7654. 30911743 10.1039/c9cp00309f

[34] T. Huang , L. Lu , Z. Ling , G. Fu , L. Zhang , H. Lu , B. Yu , H. Li , J. Lightwave Technol. 2022, 40, 5752.

[35] F. Wang , B. Nestler , J. Chem. Phys. 2021, 154, 9. 10.1063/5.0044914 33685148

[36] W. Hincheeranan , C. Chananonnawathorn , K. Duangkanya , U. Waiwijit , K. Wongpanya , R. Amarit , A. Sathukarn , S. Bamrungsap , T. Lertvanithphol , M. Horprathum , Opt. Mater. 2024, 157, 116137.

[37] J. M. De Almeida , H. Vasconcelos , P. A. Jorge , L. Coelho , Sensors 2018, 18, 1267. 29677108 10.3390/s18041267 PMC5948548

[38] H. B. Jeon , P. V. Tsalu , J. W. Ha , Sci. Rep. 2019, 9, 13635. 31541135 10.1038/s41598-019-50032-3 PMC6754453

[39] R. Carmina Monreal , S. P. Apell , T. J. Antosiewicz , Opt. Express 2014, 22, 24994. 25401533 10.1364/OE.22.024994

[40] M. Piliarik , P. Kvasnička , N. Galler , J. R. Krenn , J. Homola , Opt. Express 2011, 19, 9213. 21643175 10.1364/OE.19.009213

