# Supplementary material for: Ion‐Induced Hydrophilic Switching Enables Nanostructure Morphology Control for Superior Nanoplasmonic Sensing
Source: Small. 2025 Dec 24;22(5):e10984. doi: 10.1002/smll.202510984 (PMC12824559; doi:10.1002/smll.202510984)
Supplement: Supplementary file 1 — Supporting Information [file SMLL-22-e10984-s001.docx]

**Supporting Information**

**Ion-Induced Hydrophilic Switching Enables Nanostructure Morphology Control for Superior Nanoplasmonic Sensing**

Chia-Ming Yang^1,2,3,4,5^ Chih-Ching Ho^1,2^ Aravind Satheesh^1,2^ Chih‐Jen Yu^1^ Nikhil Bhalla^*6^

^1^Institute of Electro‐Optical Engineering, Chang Gung University, Taoyuan City 33303, Taiwan

^2^Department of Electronic Engineering, Chang Gung University, Taoyuan City 33303, Taiwan

^3^Department of Materials Engineering, Ming Chi University of Technology, New Taipei City 243303, Taiwan

^4^Department of Neurosurgery, Chang Gung Memorial Hospital at Linkou, Taoyuan 33302, Taiwan

^5^Department of Electronics Engineering, Ming-Chi University of Technology, New Taipei City 24301, Taiwan

^6^Nanotechnology and Integrated Bioengineering Centre (NIBEC), School of Engineering„ Ulster University, 2‐24 York Street, Belfast BT15 1AP, United Kingdom

Table of Contents

[XPS Results 2](#_Toc211958033)

[AFM Roughness Analysis 4](#_Toc211958034)

[Performance comparison of LSPR sensors 4](#_Toc211958035)

# **XPS Results**

F *1s* XPS spectra were acquired after C *1s* correction at 284.8 eV. The fitted F *1s* peak (Fig. S1(a)) is centered at 686.5 eV, which falls within the 686.4–686.8 eV range reported in the International XPS Database for inorganic fluorides, such as K₂SiF₆ (686.6 eV). In comparison, metallic fluorides with highly ionic M–F bonds (for example CaF₂, MgF₂, NaF) typically appear at lower binding energies around 684–686 eV due to the higher electron density around F⁻. This binding energy is distinctly lower than that of organic C–F or CF₂/CF₃ species (≈ 688–689 eV), suggesting that the detected fluorine is likely associated with Si–F/Si–O–F oxyfluoride bonding on the quartz surface formed during SF₆ plasma treatment.

Moreover, with regards to the hydrophilicity observed after SF₆ plasma treatment, the C *1s* peak of our sample appears at 284.8 eV (Fig. S1(b)), which corresponds to the standard position of adventitious carbon and was used as the reference for energy calibration. Importantly, no additional peaks at higher binding energies (≈ 288–293 eV) associated with CF, CF₂, or CF₃ functional groups were detected. In comparison, Resnik *et al.* [1] reported that SF₆ and CF₄ plasma treatments produce hydrophobic polymer surfaces when CFₓ (x = 1–3) groups are formed, with characteristic C *1s* components at 289 eV (CF), 291.3 eV (CF₂), and 293 eV (CF₃), accompanied by increased water contact angles (~106°) [1]. Since our spectrum shows only a single C *1s* peak at 284.8 eV without any CFₓ related features, we conclude that fluorocarbon species were not formed on the surface. Therefore, the observed fluorine in our sample (F 1s = 686.5 eV) is more reasonably attributed to inorganic Si–F/Si–O–F oxyfluoride bonds rather than hydrophobic CFₓ moieties based on these aforementioned works in the literature.


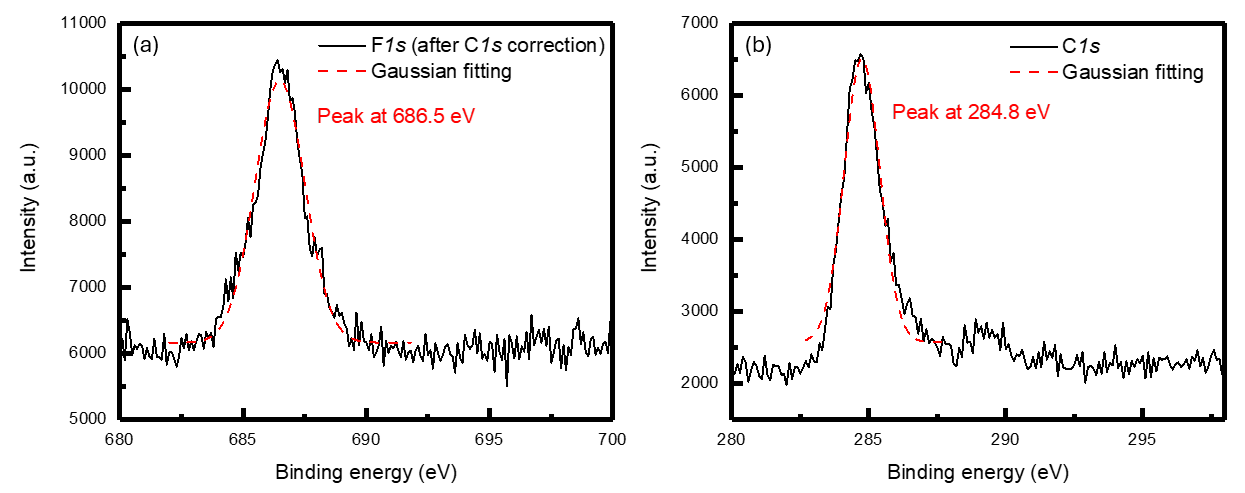


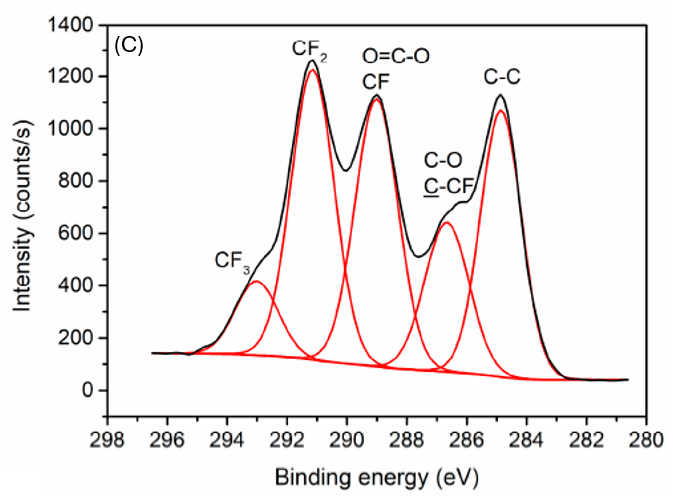


***Figure.S1*** *High-resolution XPS spectrum of the (a)F 1s and (b) C 1s for the SF₆ plasma–treated quartz glass and (c) C 1s for the SF₆ plasma–treated PET substrate. [1] Reproduced from Resnik M., Zaplotnik R., Mozetic M., Vesel A., Materials 2018, 11(2), 311.*

# **AFM Roughness Analysis**

We used a filtered SF₆ plasma. AFM shows no measurable change in roughness before/after treatment (Figure S2), indicating suppressed ion bombardment and negligible surface etching. Thus, roughness-induced effects are unlikely. To further confirm that the observed enhancement originates specifically from fluorine surface chemistry rather than generic plasma activation, additional control experiments using fluorine-free plasmas (e.g., Ar or O₂) or UV-ozone activation can be conducted in future work for direct comparison.


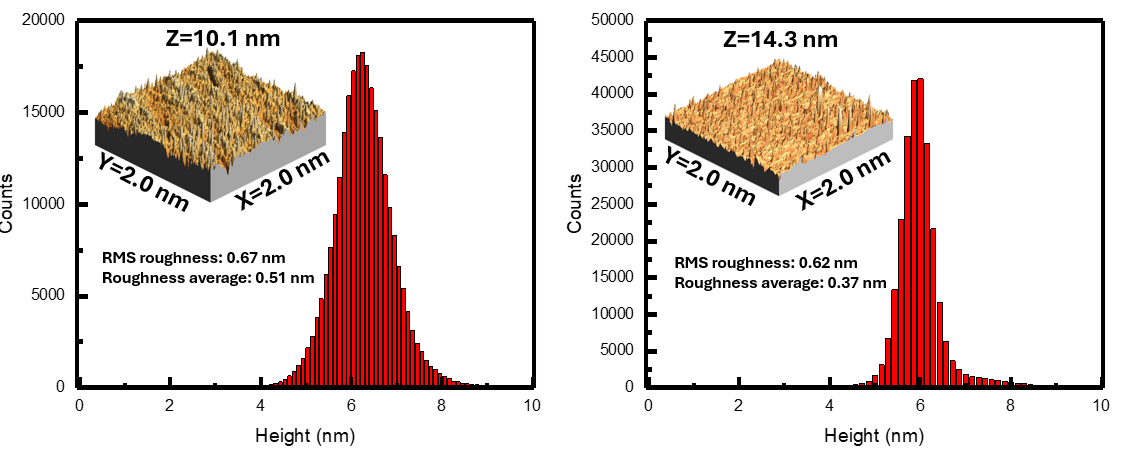


***Figure. S2*** *AFM analysis of glass (a) before SF_6_ plasma treatment and (b) after SF_6_ plasma treatment.*

# **Performance comparison of LSPR sensors**

Table.S1 summarize recent reports of lithography-free dewetting methods for fabricating Au nanoisland LSPR substrates, focusing on comparable film thicknesses (4–6 nm). As shown, our SF₆ plasma-treated glass substrate exhibits a significantly higher refractive-index sensitivity compared with other thermally dewetted Au films. The highest sensitivity of our optimized sample reached 102 nm/RIU, while the average value (95.21 nm/RIU) still surpasses those of other studies, which typically range between 40 and 80 nm/RIU for similar film thicknesses and annealing temperatures.

Regarding uniformity, three independent chips were fabricated, and five measurement points were taken per chip (N = 15 data points in total). The resulting standard deviation of the measured sensitivities was only 6.9% (6.56 nm/RIU), demonstrating excellent reproducibility. This consistent performance indicates that the SF₆ plasma pretreatment not only enhances LSPR sensitivity but also improves the uniformity of the dewetting process across the substrate.

We attribute this enhancement to two main factors:

(1) **Improved spatial control** of nanoisland formation achieved through the SF₆ plasma pretreatment, which modifies surface energy and promotes uniform dewetting.
(2) **Reduced interparticle spacing**, which enhances near-field coupling between adjacent Au nanoislands and increases the effective electromagnetic field confinement, leading to stronger plasmonic sensitivity even with smaller particle sizes.

Furthermore, our process demonstrates excellent reproducibility, as evidenced by a low standard deviation of only **6.9% (6.56 nm/RIU)** across 15 measurements (three chips × five points each). This confirms that the plasma-assisted approach not only enhances sensitivity but also enables controllable and highly uniform nanostructure fabrication, offering an effective and scalable route for robust LSPR sensing substrates.

Table. S1 Performance comparison of LSPR sensors fabricated by Au thin film dewetting

| **Method** | **Year** | **Substrate** | **Particle size  (nm)** | **Interparticle distance  (nm)** | **Sensitivity  (nm/RIU)** | **Uniformity (Sensitivity deviation)** | **Ref.** |
| --- | --- | --- | --- | --- | --- | --- | --- |
| Au 4 nm dewetting 650℃ 1 min | 2025 | SF_6_ plasma treated glass | 14.78 | 14.96 | 102 (highest one) 95.21 (Average) | 6.9% (6.56 nm/RIU) (N=chip*3 x point*5 = 15) | **This work** |
| Au 4 nm dewetting 560℃ 3 hr | 2019 | SiO2 | 24.7 | 29.2 | 43.17 | N/A | [2] |
| Au 6 nm dewetting 500℃ 2hr | 2024 | glass | 68 | ≈ 90 | 60.17 | ≈ 8.2% (4.51 nm/RIU)  (N=3 chips) | [3] |
| Au 6 nm dewetting  900℃ 5 min | 2017 | glass | 39.9 | N/A | 80.7 | N/A | [4] |
| Au 6 nm dewetting 600℃ 1hr | 2019 | SiO2 | 41.9 | N/A | 59.82 | N/A | [5] |
| Au 4 nm dewetting 630℃ 10.5hr | 2021 | glass | 51.5±21.4 (minor axis) - 70.9±31.7 (major axis) | N/A | 72 | N/A | [6] |
| Au thin film dewetting 500℃ 2hr | 2025 | glass | 30 - 90 | 20 - 150 | 36.118 - 92.165 | 5.14%   ($\Delta\lambda$ for 5 chips) | [7] |

**Supplemental References**

[1] M. Resnik, R. Zaplotnik, M. Mozetic, and A. Vesel, "Comparison of SF6 and CF4 plasma treatment for surface hydrophobization of PET polymer," *Materials,* vol. 11, no. 2, p. 311, 2018.

[2] N. Bhalla, A. Jain, Y. Lee, A. Q. Shen, and D. Lee, "Dewetting metal nanofilms—Effect of substrate on refractive index sensitivity of nanoplasmonic gold," *Nanomaterials,* vol. 9, no. 11, p. 1530, 2019.

[3] W. Hincheeranan *et al.*, "A study of multiple solid-state dewetting of sputtered Au ultra-thin films for chip-based LSPR sensor applications," *Optical Materials,* vol. 157, p. 116137, 2024.

[4] H. T.-H. Lin, C.-K. Yang, C.-C. Lin, A. M.-H. Wu, L. A. Wang, and N.-T. Huang, "A large-area nanoplasmonic sensor fabricated by rapid thermal annealing treatment for label-free and multi-point immunoglobulin sensing," *Nanomaterials,* vol. 7, no. 5, p. 100, 2017.

[5] J. M. De Almeida, H. Vasconcelos, P. A. Jorge, and L. Coelho, "Plasmonic optical fiber sensor based on double step growth of gold nano-islands," *Sensors,* vol. 18, no. 4, p. 1267, 2018.

[6] N. M. Figueiredo, R. Serra, and A. Cavaleiro, "Robust LSPR sensing using thermally embedded Au nanoparticles in glass substrates," *Nanomaterials,* vol. 11, no. 6, p. 1592, 2021.

[7] T. Lamtha *et al.*, "Solid-state dewetting sputtered ultra-thin Au films for LSPR sensing chip toward African swine fever virus detection," *Sensors and Actuators A: Physical,* vol. 382, p. 116165, 2025.
